# Imagery Rescripting of Early Traumatic Memories in Social Phobia

**DOI:** 10.1016/j.cbpra.2011.03.002

**Published:** 2011-11

**Authors:** Jennifer Wild, David M. Clark

**Affiliations:** Institute of Psychiatry, King's College London

**Keywords:** imagery rescripting, imagery, trauma memory, social phobia

## Abstract

Negative self-images appear to play a role in the maintenance of social phobia and research suggests they are often linked to earlier memories of socially traumatic events. Imagery rescripting is a clinical intervention that aims to update such unpleasant or traumatic memories, and is increasingly being incorporated in cognitive behavioral therapy programs. In previous research, we have found that imagery rescripting was superior to a control condition in terms of its beneficial effects on negative beliefs, image and memory distress, fear of negative evaluation, and anxiety in social situations. In this article, we describe our imagery rescripting procedure. We consider the importance of updating negative imagery in social phobia, the theoretical basis for imagery rescripting, directions for future research, and how to conduct imagery rescripting, including potential problems and their solutions.

## Research Highlights

► Imagery rescripting is a clinical intervention that aims to update distressing memories. ► We describe our imagery rescripting procedure of early traumatic memories in social phobia. ► The procedure consists of a cognitive restructuring component then 3 stages conducted in imagery.► Consideration is given to how and why imagery rescripting works. ► Potential problems and their solutions are also presented.

In social situations, patients with social phobia often experience distorted, negative images or impressions of how they fear they will come across to other people (i.e., Hackmann, Clark, & McManus, 2000; Hackmann, Surawy, & Clark, 1998). Research has reported that the negative images/impressions are often linked in meaning and content to early socially traumatic (embarrassing/humiliating) events clustered around the onset of the disorder (Hackmann et al., 2000). In the treatment of social phobia, it is necessary to update these negative images because they maintain social anxiety. They cause patients to feel more anxious and to perform less well than when they hold benign imagery in mind (e.g., Hirsch, Clark, Mathews, & Williams, 2003). Further, the negative imagery prevents patients from disconfirming their social fears, which can include, for example, a fear of running out of things to say or of blushing, of people noticing and then concluding that they are inadequate or incompetent.

Negative imagery appears to maintain social fears for a number of reasons. First, patients believe their negative self-images are a true reflection of how they come across to other people. They therefore think they come across much worse than they actually do, which reinforces rather than disconfirms their perception of performing inadequately. Second, negative imagery motivates patients to use safety-seeking behaviors, which can interfere with their social performance and make them appear less interested in other people than they really are (Alden & Taylor, 2004; Clark & Wells, 1995; Hirsch, Meynen, & Clark, 2004; Rapee & Heimberg, 1997). Third, negative self-imagery blocks positive interpretation bias (Hirsch, Mathews, Clark, Williams, & Morrison, 2003). This means when faced with an ambiguous social cue, such as a smile from a conversational partner, patients with social phobia are unlikely to make a positive interpretation about the smile and so miss opportunities to benefit from the very feedback that could help them to reevaluate their fears and reduce their anxiety. Fourth, negative imagery facilitates selective retrieval of negative memories (Stopa & Jenkins, 2007) and there is evidence that judgments about the future probability of an event are influenced by the accessibility in memory of past instances (Tversky & Kahneman, 1974).

Several cognitive behavioral therapy (CBT) programs for social phobia include present-focused techniques to correct distorted self-images, such as videofeedback, surveys, and behavioral experiments. These techniques are employed almost immediately in cognitive therapy for social phobia (Clark, 1999) because of the pivotal role negative imagery has in maintaining patients’ social fears, avoidance, and anxiety. Given that the images are often linked in meaning and content to distressing memories, it also makes sense to treat the origins of the images, particularly if patients continue to experience negative imagery following intervention with these present-focused techniques.

Imagery rescripting describes a set of related therapeutic procedures that focus on changing unpleasant memories (Stopa, 2009). The procedure is also known as imagery with rescripting (e.g., Arntz & Weertman, 1999), and throughout this paper, we use these terms interchangeably. Imagery rescripting techniques have been used as major components of CBT programs for borderline personality disorder (Giesen-Bloo et al., 2006), bulimia (Cooper, Todd, & Turner, 2007), and posttraumatic stress disorder arising from childhood sexual abuse (Smucker & Neiderdee, 1995). Turning to social phobia, Clark and colleagues have recently incorporated imagery rescripting techniques into their cognitive therapy program, particularly for patients who have made only modest improvements with present-focused techniques. A recent trial (Clark et al., 2006) found that this integrated cognitive therapy program was superior to exposure therapy, and the authors speculated that the overall beneficial effects of cognitive therapy for social phobia were partly due to the use of imagery rescripting. To formally test the role of imagery rescripting per se, Wild, Hackmann, and Clark (2007, 2008) conducted two studies that assessed the effects of imagery rescripting alone in unselected populations of patients with social phobia. Wild et al. (2007) reported pre- and post-rescripting results in 14 patients with social phobia with whom they developed the approach. Imagery rescripting alone was associated with significant improvements in patients’ negative social beliefs, the vividness and distress of their image and early memory, and in self-report measures of social anxiety. Wild et al. (2008) then compared a session of imagery rescripting with a control session in which images and memories were explored without being updated. Measures were taken before each session and 1 week later. The imagery rescripting session was associated with significantly greater improvement in negative beliefs, image and memory distress and vividness, fear of negative evaluation, and anxiety in feared social situations.

In this paper we describe in detail our procedure of imagery rescripting for social phobia, which includes a cognitive restructuring component, and which demonstrated effectiveness in Wild et al. (2007, 2008). We first present the theoretical basis for the technique, then a description of how to conduct it, followed by clinical examples, how to address potential problems, and directions for future research.

## Theoretical Basis

The theoretical basis for employing imagery rescripting in the treatment of patients with social phobia lies in the link between their recurrent imagery in the present and their past socially traumatic events. We define a socially traumatic event to be an extremely unpleasant social event in which the individual experiences intense anxiety and perceives concurrent ridicule or rejection by others, such as being bullied at school, performing poorly in a meeting at work, and believing that colleagues or peers are silently ridiculing the individual, or being humiliated for exhibiting signs of anxiety, for example. These events go beyond feeling as though a social performance situation has gone badly and include perceptions of humiliation, ridicule, intense criticism, or rejection.

Hackmann et al. (2000) reported that recurrent imagery and past socially traumatic events were often linked in theme and content. In fact, the recurrent images tend to be visualizations of aspects of memories for past socially traumatic events. That is, the images are derived from past memories. These images appear to be triggered in different social situations by cues that match the original event in some way. Like intrusive images in posttraumatic stress disorder (PTSD), images in social phobia heighten anxiety and remind the patient of past danger. The patient approaches current social situations as if the contingencies that appeared in the past event are still relevant, typically expecting people to respond to them in the same way as they did in their memory of the socially traumatic event. Just as the memory images have similar cues to the past event, they also carry a similar meaning to the original memory, an “encapsulated belief” that captures the meaning of both (Wild et al., 2008).

Wild et al. (2007, 2008) reported that sometimes patients recalled catastrophic outcomes linked to their earlier memory that may not have happened in the way they had thought. This was discovered when patients relived their earlier event with the therapist as part of the imagery rescripting session. For example, one patient had a recurrent image of looking as though he was curled up in a shell, frightened, and incapable. This linked to a memory of when he was 16 years old in sixth form (i.e., Grade 11):

A group of children upon seeing the patient in the canteen said, “Hey, there's Katy's brother. They don't look related.” The patient blushed, felt frightened and diminished. His sister was popular and outgoing. When he heard the comment, “They don't look related,” he interpreted this as meaning that he had failed to meet their expectations and they were rejecting him. Thinking this, he quickly left the canteen. However, there was no clear evidence at the time that he was being rejected and there were many other instances when he had good, protracted interactions with these children. Nevertheless, his encapsulated belief captured the essence of social rejection: “I'm odd and a failure, incapable, and less than what people expect. People will see I am less than expected, and reject me.”

For other patients, the early rejection did occur but they are no longer rejected in a similar manner as adults. However, their encapsulated belief retains the much earlier self-impression. Therefore, when updating the earlier memory and the recurrent imagery, the imagery rescripting session must address the encapsulated belief, which links the two. For this reason, we conduct cognitive restructuring of the encapsulated belief prior to our imagery with rescripting process.

Cognitive restructuring aims to challenge the patient's encapsulated belief and to ready an adult perspective that they may draw upon during the imagery rescripting phase. Cognitive restructuring alone can often produce an intellectual understanding that the perceived contingencies from the past do not apply to the present. However, it is our experience that incorporating this information into the memory through the use of imagery rescripting is often needed to produce emotional, as well as intellectual, change. When discussing therapy for patients with borderline personality disorder, Arntz and Weertman (1999) make a similar observation, suggesting that experiential methods, such as imagery rescripting, are more effective than verbal reasoning alone for modifying problematic negative beliefs and memories related to childhood. In our work with PTSD, we have similarly noted that the intellectual shifts that occur with cognitive restructuring alone can be limited in their impact but can be much enhanced by inserting the new information derived from cognitive restructuring into the trauma memory during a planned imaginal reliving of the traumatic event (see Ehlers, Hackmann, & Michael, 2004, for an extended discussion). Teasdale (1993) has suggested that part of the reason for the greater impact of imagery may lie in its ability to activate multiple representations that are better at accessing implicational meaning (Teasdale).

The imagery rescripting procedure that we use includes imaginal reliving (Foa & Rothbaum, 1998), and is similar to the three stages described by Arntz and Weertman (1999). While other clinical researchers have described imagery methods (e.g., Cooper et al., 2007; Edwards, 1990; McGinn & Young, 1996; Smucker & Neiderdee, 1995; Young, 1994), none have been described using these methods for patients with social phobia and their distinctive images. We drew on Arntz and Weertman in compiling our procedure because their method included a stage that involved taking a compassionate stance towards the younger self. Given the difficulty patients with social phobia often have in spontaneously accessing compassion for themselves following socially traumatic events, this seemed likely to be an important component. In Stage 1, similar to Arntz and Weertman, we had patients relive the socially traumatic event from the age at which it occurred. In Stage 2, they relive the event again but from an adult observer perspective, observing their younger self as the event unfolds. In Stage 3, they relive the event again from the age at which it occurred. On this occasion, their adult self is with them and offers updated information—derived from cognitive restructuring—about how they come across now, takes a compassionate stance towards their younger self, and, if necessary, can intervene.

Our imagery rescripting procedure differs from Arntz and Weertman (1999) in three ways. First, we have a cognitive restructuring phase prior to the imagery rescripting procedure. Second, in Stage 2 of the imagery rescripting process, we ask the patient to relive the incident from the adult's perspective, but do not specifically ask the adult to intervene at that time, although if they wish to, they may. Arntz and Weertman do ask the adult to intervene in both Stages 2 and 3. We chose to have the adult self intervene in Stage 3 only. In this stage, as described by Arntz and Weertman, the child or younger self relives the incident again and the adult self intervenes. The younger self can ask the adult for further interventions and for what they need, and then receive this. It was our impression from our pilot work that it was the child or younger self that needed to experience the intervention and that this would occur when they were reliving the event from that perspective (i.e., Stage 3) rather than from the adult perspective (i.e., Stage 2). Third, we do not discuss the stages after each one but rather move from one stage to the next with the patient keeping their eyes closed for the duration of the imagery procedure, which takes approximately 45 minutes. Table 1 shows a summary of the different stages of the full imagery procedure.

Thus, our imagery rescripting session includes a number of potentially therapeutic techniques: a period of cognitive restructuring, imagery with rescripting, which involves repeated evocation of the socially traumatic memory (in Stages 1, 2, and 3), corrective information inserted into the memory image (in Stage 3), and compassionate imagery (in Stage 3). The cognitive restructuring allows the patient to identify a convincing, intellectual argument against the encapsulated belief. Repeated evocation of the socially traumatic memory in a planned and controlled way helps to lead to its reevaluation (Foa & Rothbaum, 1998). Inserting corrective information, such as “blushing is not a sign of failure,” into the socially traumatic memory ensures that adaptive rather than negative interpretations are assimilated into the memory image. Compassionate imagery in which the patient pictures their adult self warmly embracing their younger self, for example, may enhance the patient's feeling of being accepted, a central concept in social phobia. Finally, conducting much of the procedure in imagery may be beneficial: it may engender the experience of having had a concrete experience (Epstein, 1994). Lang (1977, 1979) suggests that the physiological, emotional, and behavioral responses activated during imagery are similar to what is activated in real scenarios. Drawing on research in neuroscience, we see that imagery of movement, for example of the hands, toes, or tongue, uses the same cortical circuitry (e.g., Schnitzler, Salenius, Salmelin, Jousmäki, & Hari, 1997) and results in the same motor cortical activation (e.g., Ehrsson, Geyer, & Naito, 2003) as actually moving these parts of the body. This suggests that at the level of brain activation, imagining movement is similar to actually doing it. While imagery rescripting is much more than imagining movements, it is possible that the physiological, emotional, and behavioral responses it generates feel as real as actually having had these experiences, which may be therapeutic for clients (Wild et al., 2008).

## When to Use Imagery Rescripting for Patients With Social Phobia

Imagery rescripting, as it has been developed and evaluated in the context of social phobia, is an intervention with the primary aim of updating the earlier memory from which patients’ negative imagery stems, and the meaning linking the recurrent negative image and memory. For this reason, it is intended for patients who experience negative imagery that is linked to a past socially traumatic event, and whose response to standard, present-focused techniques to correct distorted self-images has been relatively modest. It should be noted that while many patients with social phobia report negative imagery, and for many, this is linked to an identifiable event in the past (i.e., Hackmann et al., 2000), some patients experience negative imagery, which appears to be unrelated to an earlier event. For these patients, the standard present-focused imagery modification techniques, such as videofeedback, behavioral experiments, and surveys, will likely be beneficial when offered as part of CBT programs for the disorder. We would recommend delaying deploying imagery rescripting during an integrated cognitive therapy for social phobia program until the patient has attended a minimum of four sessions of therapy. At that point, the patient will have had time to experience the benefits of videofeedback and some behavioral experiments, which they may then draw upon in the cognitive restructuring phase of the procedure.

## Imagery Rescripting Session

### Identifying the Recurrent Image, the Linked Memory, and the Encapsulated Belief

As described above, our imagery rescripting session begins with a period of cognitive restructuring followed by three stages of imagery rescripting. At the beginning of the session, it is necessary to identify the patient's recurrent image, the memory it is linked to, and the encapsulated belief that captures the meaning of both. To identify patients’ recurrent imagery, we draw on Hackmann et al. (2000) and ask: “I'd like to talk to you about some of the things that go through your mind when you get anxious in social situations. Usually when people are very anxious a mixture of thoughts and images or fleeting pictures go through their minds. I'm especially interested in any pictures or images you have popping into your mind when you're anxious. Do you have any spontaneous images when you are anxious in social situations?” We then ask patients to close their eyes and to recreate the image, then describe it. To determine the meaning of the image, we ask patients: “What is the worst thing about the image? What does it mean about you as a person?”

To identify the memory linked to the image, we ask patients when they first remembered feeling the way they did in their image. We then ask them to close their eyes, get a clear image of the event associated with that feeling and describe the image. Patients are encouraged to describe the event in the present tense, as though it is happening again. To determine the meaning of the memory, we ask patients: “What is the worst thing about the memory? What does it mean about you as a person?” We then summarize the meaning of the image and memory and ask patients to give one or two sentences that would “encapsulate” the meanings. One patient, for example, phrased the encapsulated belief linking her image and memory as “I'm an outsider and always will be. People will reject me or laugh at me because I'm not like them.” Her recurrent image was of looking awkward, jittery, twitchy, and speaking in garbled sentences. This was linked to a memory of when she was 13 years old and a group of children at her school cornered her against a wall. They made fun of her for the way she was twitching and her inability to speak. She thought she would be attacked in front of all the other children and it would be humiliating (Wild et al., 2008).

### Cognitive Restructuring

When we have identified the encapsulated belief, we take a belief rating and begin cognitive restructuring. Typically this lasts 30 to 45 minutes. We ask the patient to outline the evidence they had for their encapsulated belief at the age at which their socially traumatic event occurred. We then help them to challenge the belief with evidence they have accumulated as an adult, some of which they will have gained through conducting behavioral experiments and videofeedback as part of their cognitive therapy for social phobia. We use the whiteboard to write out evidence for the encapsulated belief and the alternatives, working with the patient to challenge the meaning of the early event and its implications for the present. This may include, for example, thinking of all the reasons why children bully other children and what this says about the bullies rather than the patient. Patients may also be encouraged to think of examples in which they were not rejected then or now. In essence, the therapist helps the patient to distinguish between what happened when they were a young child/teenager and what happens now that they are an adult in order to help them to see the event as a time-limited experience without implications for their present or future, so that an adult perspective can be readied and drawn on in the imagery rescripting procedure (Wild et al., 2008). Below we provide two examples of evidence for and against encapsulated beliefs garnered through cognitive restructuring. One is an example of a patient whose worst fears did *not* occur: he was not actually rejected during his socially traumatic event but perceived that he was. The other case is an example of a patient whose worst fears *did* occur: she blushed, and was humiliated as a result.

#### Clinical Example: Worst Fears Did Not Occur

Rob, briefly described above, had a *recurrent image* of looking as though he was curled up in a shell, frightened, and incapable. This linked to a *memory* of when he was 16 years old in sixth form (i.e., Grade 11). A group of children saw him in the canteen and said, “Hey, there's Katy's brother. They don't look related.” Rob blushed, felt frightened and less than expected. He left the canteen, believing the other children had rejected him. Because his sister, Katy, was popular and outgoing with a lot of friends, he believed that her friends would expect him to be extroverted, socially competent, and as popular as she was. When he heard the comment, “They don't look related,” he interpreted this to mean that they were judging him negatively, that he did not measure up to what they expected, and that they thought he was odd and socially incapable. Because he had blushed and had felt frightened, he also thought he was a failure. The encapsulated belief linking his imagery and memory was: “I'm odd and a failure, incapable, and less than what people expect. People will see I am less than expected, and reject me.” Table 2 summarizes his evidence for the belief and the alternatives he generated with his therapist during the cognitive restructuring phase.

#### Clinical Example: Worst Fears Did Occur

Megan came to therapy when she was 30 years old. She had a *recurrent image* in social situations of looking as though she was blushing scarlet red with a sense that people were laughing at her and pointing at her, the way her ex-boyfriend did, as though she were inferior. This linked to a *memory* that occurred when she was 18 years old and at university. One evening, she was chatting to her boyfriend, Jeff, in her room in residence when his friend, Neil, came over. He used her toilet, clogged it, and left it full of faeces. Megan used the toilet after him and was surprised at the mess. She made a mental note to clean it later, and decided to leave it for the time being. She did not say anything. She closed the door, left Jeff and Neil chatting in her bedroom, and went to the communal kitchen. When she went back to her room about 10 minutes later, her boyfriend had gone to the bathroom. He had seen the mess. He did not believe that she had not made it. She started to blush. He dragged her in front of a mirror and said, “Omigod, I cannot believe how much you're blushing!” Megan did not open her eyes to look in the mirror. She felt humiliated. Then they all went into the kitchen where Neil joked about it in front of other people.

The encapsulated belief linking her image and memory was: “I am inferior to other people, people will see this and reject me.” As the examples in Table 3 show, the cognitive restructuring phase allows patients to come up with new information and alternatives to what they had perceived to be evidence supporting their encapsulated belief. The alternatives draw on new information they have gained in therapy.

### Imagery Rescripting

Following the cognitive restructuring phase, we then move into the imagery rescripting procedure. We give patients the following rationale:

We've seen that a traumatic event led you to develop certain beliefs about yourself and to feel as though people will respond to you in the present in a similar way to what happened in the past. It is like you have been processing the present on the basis of the restricted information that you had in the past. At the time you were a child/younger person and you did not have access to current/adult information. We have seen that as an adult, you do not get rejected, and the world does not expect you to be perfect.

We've seen that although the memory was painful, you were not actually rejected, although it very much felt like that at the time (or you were rejected on that occasion but are no longer rejected now).

We need to update the memory to bring in this new information that we have discovered.

The way we do that is to revisit the memory again. For you to tell it in the first person present-tense as though you are the (for example) 18-year-old girl again. And then to bring in the new information as an adult. To see (for example) 30-year-old Megan intervening. This may involve talking to (for example) 18-year-old Megan and telling her what you know now, you may also feel like intervening in another way, perhaps talking to the children who pointed you out.

The aim of the procedure is to update the memory so that it is no longer an event which colors your present, so that you can accurately process the present as it is really happening.

I may prompt you as we go along. Do you have any questions?

#### Imagery Rescripting: Stage 1

In Stage 1, patients are asked to close their eyes and to talk the therapist through the memory at the age at which it occurred. This phase is similar to imaginal reliving of traumatic memories in CBT for PTSD (e.g., Ehlers & Clark, 2000; Foa & Rothbaum, 1998). The patient talks through the event in the present tense with eyes closed. Below is a transcribed example of Phase 1 with Megan, the patient whose boyfriend humiliated her for blushing.

Therapist: When you're ready, sit comfortably, close your eyes and take yourself back. You're 18 years old and you're in your halls at university. You are chatting with your boyfriend, Jeff, in your room…. Tell me what happens, take me through what happens as if it's happening right now.

Megan: Um okay, so I am in my room, talking to Jeff, and there's a knock at the door. I open it. It's Neil. He barges past me right into my room, he slaps Jeff on the shoulder. “Hey mate,” he says. The two of them start joking and messing about, talking about football or something. I walk to my bed, I sit down next to Jeff. Neil gets up almost immediately, he goes into the bathroom. He's in there for a while. I am chatting to Jeff about a film we might see later on. Neil comes out and takes over the conversation. So, I get up, I go to the bathroom. I can't believe what I see in there. It is disgusting. Neil has not flushed the toilet, it's clogged and it looks like there's poo everywhere. “I can't deal with this now,” I think to myself. I decide I'll clean it up later. I leave the bathroom and then my room. I just want to get away from the two of them together. I putter about the kitchen for about ten minutes, then I head back to my room. Neil and Jeff are still in there. They are laughing loudly. As I walk in, Jeff says, “Megan, why did you leave the bathroom in such a mess?” Then I, I um, um . . .

Therapist: That's great, Megan, you're doing a great job. So Jeff says to you, “Why did you leave the bathroom in such a mess?” Just stay with what's happening, what happens next?

Megan: I look at Jeff in the eyes and I tell him, “I didn't do it. It wasn't me.” They laugh. I can feel my face getting really hot. I feel hurt. Jeff should believe me. I say again, “I didn't do it.” He pulls me by the arm and drags me in front of the mirror. I close my eyes. I hear him say, “Omigod, I cannot believe how much you are blushing!” I yank my arm away and get out of my room as quickly as possible. They follow me to the kitchen, laughing. We start making dinner and Neil keeps going on about the mess in the bathroom and how much I was blushing. I want the world to swallow me up. I feel so ashamed and hurt. I can't believe Jeff, my boyfriend, chooses to believe his friend over me.

#### Imagery Rescripting: Stage 2

In Stage 2 of the imagery rescripting procedure, clients relive their socially traumatic event again, but this time they observe what happens to their younger self as if they are in the room watching the events unfold. Below is Megan's transcript of Stage 2.

Therapist: You are doing a great job, Megan. Now, keep your eyes closed. We're going to move into the next phase of this procedure. What I would like you to do now is to talk me through the event again, but this time I want you to tell it to me as though you are observing what is happening, as though you are in the room, watching the events unfold. So, this would mean talking me through the event in the third person. “I see Megan in her room, she is chatting to her boyfriend . . .” Tell me what you see.

Megan: Okay. Megan is in her room. She is chatting to her boyfriend about a movie they are thinking of seeing. She hears a knock at the door and I see her go and open it. It's Neil. She doesn't like Neil but she opens the door. He barges past her and right into her room. She goes and sits next to Jeff. Neil gets up and goes to the bathroom. I see her and Jeff chatting again. Then Neil comes out of the bathroom. He overtakes the conversation and he and Jeff get all chummy and exclusive. I see Megan go into the bathroom, and omigod the mess she has to deal with! There is poo everywhere. She leaves the bathroom, deciding not to take it up with Neil just then. She doesn't want to embarrass him. She leaves the lads in her room and goes to the kitchen. After about ten minutes, she returns. I see Jeff and Neil being immature. I see Jeff blame Megan for the mess in the bathroom. This is ridiculous! She didn't even make that mess! She was just trying to be kind and considerate and she gets blamed for it! She was just trying to do the sweetest thing and her boyfriend is being a complete jerk.

Therapist: That's right. He is being a jerk. And what happens next? What do you see happening next?

Megan: Omigod! Jeff grabs Megan by the arm and drags her in front of a mirror. This is so wrong! He says, “Omigod, I cannot believe how much you are blushing!” That is so cruel. What a jerk. Megan doesn't open her eyes, she doesn't look in the mirror. She turns around and leaves her room. The boys follow and they head to the kitchen, where they keep teasing her. This is so wrong! They are such idiots.

#### Imagery Rescripting: Stage 3

In Stage 3 of the imagery rescripting procedure, clients relive their socially traumatic event again at the age at which it occurred, but this time, their wiser older self is with them and can intervene, offer compassion, or new information to update the event and its implications.

Therapist: Good work, Megan. We are almost done. Now keep your eyes closed. We are going to go through this one more time. This time, I want you to talk me through it again as if you were 18-year-old Megan and it is happening right now. But this time, your wise 30-year-old self is in the room with you. She has all the information you have learned in therapy and she can intervene if you want her to, she can talk to Jeff and Neil or do anything else that feels helpful and right in this situation. Are you ready? Okay, take me back to your halls of residence, you are in your room talking to your boyfriend.

Megan: I am in my room with Jeff. We are talking about a film we want to see. I hear a knock at the door. I get up and Neil walks right in, past me, and straight to Jeff. They start talking about something I don't really understand or care about. I sit on my bed. Neil goes into the bathroom. I mention the film again to Jeff. Neil comes out of the bathroom and they start messing about again, so I go into the bathroom. There is so much mess in there. I can't believe it. I don't know what to do. I decide I will talk to Neil about it later and clean it up when he has gone. I come out of the bathroom and Neil and Jeff are still talking so I go to the kitchen. I am there for about ten minutes. Then I go back to my room. I can hear Jeff and Neil laughing as I am getting close to my room. When I walk in, Jeff accuses me of making a mess in the bathroom. I can't believe, it's not even my mess! It's not my mess.

Therapist: That is right. What do you feel inclined to do?

Megan: I want to tell him to grow up.

Therapist: So, see older Megan saying this to Jeff.

Megan: Older Megan says to Jeff, “Hey, if you can't believe me that's your problem, not mine. Why do you have to act so immature when you're with Neil? It's like you downplay how you feel about me. You're difficult to be with and dumping you will be one of the kindest things I ever do for myself. After we break up, I meet someone who really values me and puts me before their friends. That's how relationships are supposed to be. They're not about humiliating the person you supposedly care about.”

Therapist: How does Jeff respond?

Megan: He looks sheepish, kind of sorry. He says he doesn't want me to take it personally.

Therapist: And what happens next?

Megan: He drags me in front of the mirror and points at my blushing. But I don't open my eyes.

Therapist: What do you feel inclined to do?

Megan: I kind of want to open my eyes, but first I want to tell Jeff that blushing is not a big deal. Everyone does it.

Therapist: So, see yourself saying this to Jeff.

Megan: So older Megan says to Jeff, “You know, blushing is not such a big deal. I have even seen you blush. I just don't point it out because that would be hurtful and who cares if you blush? That's not the most important thing in life. You should be ashamed of how you have treated your girlfriend, you were lucky to be with her for as long as you were. When she dumps you, she'll start to feel much better about herself and you'll be the one who loses out, mate.”

Therapist: And how does Jeff respond?

Megan: He kind of laughs but then stops and looks sheepish. I see him take Megan's hand. He says sorry. He says he is sorry he did not treat her well. He says he was immature and not ready to have the kind of relationship she wanted, so he put his friends first.

Therapist: And what about this mirror?

Megan: Older Megan asks younger Megan to open her eyes and look in the mirror.

Therapist: And what does she see?

Megan: She sees . . . she sees that she is blushing but it is just a little pink and nothing more. She sees a calm and thoughtful woman in the mirror with a smiling face, someone at the start of their life who is going to get out of this crap relationship and meet someone who really cares about her.

Therapist: Is there anything that Megan needs to do or say?

Megan: She needs to know that she is really loved.

Therapist: Can you tell her in your own quiet way.

Megan: Yes.

Therapist: And how does she respond?

Megan: She fills up with bubbly light and she feels light and confident and loved.

Therapist: Is there anything else that she needs to do or say?

Megan: Um, she needs to know that everything is going to work out for the best. She is going to overcome the insecurities Jeff made her feel.

Therapist: Can you say that to her?

Megan: Yes, I am telling her. . . . She is smiling.

Therapist: Is there anything else she needs to do or say?

Megan: She feels good. There's nothing else.

Therapist: Okay, when you are ready, bring your attention back to this office. Take your time and open your eyes.

When the third stage is complete, we ask patients to open their eyes. We ask them how they feel, and how the memory feels to them now. We then take a belief rating for the encapsulated belief.

## Potential Problems

### Intervening in Stage 2

Normally in Stage 3 of the imagery rescripting procedure, patients intervene in imagery, telling off the bullies or other offending individuals and standing up for their younger self. However, during Stage 2, when they observe their younger self experiencing the socially traumatic event, they may simultaneously realize that the event has implications about the personalities of the other people involved rather than their own, and they may spontaneously intervene in imagery in this phase. If this occurs, continue as if it is Stage 3 with questions such as, “Is there anything else that you need to do or say?”

### Reliving the Event in the Past Tense

Sometimes the patient re-tells their event in the past tense or begins telling it in the present tense and slips into the past tense. Remind them to stay in the present tense by interjecting in the present tense. For example, “…. So Megan *is* in her room. She *hears* a knock at the door…”

### Imagery Involving Violence

By the end of Stage 3 it is important to ensure that patients feel that there is nothing else that they need to do or say, that their younger self has received compassion, and that they feel calm and comforted before finishing the procedure and bringing their attention back to the present. Arntz and Weertman (1999) suggested that imagining the use of weapons may help the patient to feel stronger when they imagine their intervention. We have not found this to be necessary in patients with social phobia. However, it is not in principle ruled out. There may be instances when imaging such retribution is helpful, but obviously one needs to be clear that it is just imagining.

### Multiple Traumatic Memories

The patient may have multiple socially traumatic memories and it may be unclear which one to focus on. Ask the patient which memory they find most distressing and initially work with that memory. Since many of the memories will likely have a similar theme (i.e., rejection or humiliation), the work completed with one should generalize to the others. However, if this does not occur, then the therapist may need to rescript one or two other memories in future sessions.

## Directions for Future Research

Our imagery rescripting procedure involves several therapeutic interventions, such as cognitive restructuring, repeated evocation of the socially traumatic memory, and compassionate imagery. It is unclear which are most effective and whether all add to the value of the procedure. In our extensive piloting of the intervention, it appeared that each component was important, but this has not been shown empirically. A component analysis study is needed to clarify the relative importance of each component. Future research is also needed to determine the long-term benefits of imagery rescripting. Our initial research (i.e., Wild et al., 2007, 2008) has shown benefits at 1-week follow-up. However, it is necessary to determine that the gains with imagery rescripting are maintained for longer periods of time.

Our complete imagery rescripting intervention requires around one and half hours of therapist time. Future research could aim to shorten this, possibly by having patients complete a self-study module that helps them to identify their recurrent image, their linked memory and encapsulated belief on their own, with specific prompts to help them to reevaluate it. It is also possible that the imagery rescripting session could be completed in a modular or Internet format and future research could determine if this is possible and best methods to do this.

## Conclusion

Addressing negative self-imagery with present-focused techniques is a key component of many CBT programs for social phobia. However, the recurrent negative self-images that patients with social phobia report are often linked to earlier socially traumatic events. These events go beyond feeling as though a social performance situation has gone badly; rather, they include experiences of intense anxiety in which the patient perceives humiliation, ridicule, extreme criticism, or rejection—the very features that make them socially traumatic and indicate the potential utility of imagery rescripting for early memories in social phobia. Imagery rescripting may be useful for other disorders for which recurrent negative imagery has been linked to earlier unpleasant events, such as agoraphobia (e.g., Day, Holmes, & Hackmann, 2004), and health anxiety (e.g., Muse, McManus, Hackmann, Williams, & Williams, 2010).

The imagery rescripting procedure we have described in this paper is one in which we have evaluated in former research (i.e., Wild et al., 2007, 2008). It includes a phase of cognitive restructuring followed by three stages of imagery rescripting, drawing on the imagery rescripting procedure described by Arntz and Weertman (1999). Our procedure differs from Arntz and Weertman in that we include a component of cognitive restructuring followed by three stages of rescripting in which intervention usually only occurs in Stage 3. The aim of our procedure is to update the socially traumatic memory and the meaning linking the negative image and memory. It is indicated for patients who have made only modest improvement following standard present-focused techniques, such as videofeedback and surveys, in the CBT treatment of social phobia, and whose recurrent negative image links to an identifiable earlier event. Imagery rescripting for social phobia is intended to be offered as part of a CBT treatment package for the disorder. In our studies, the procedure was applied judiciously by therapists who had extensive prior experience with cognitive therapy. It is unclear how much prior general training in cognitive therapy is required for the effective delivery of the intervention, but this could be clarified with future research.

## Figures and Tables

**Table 1:** Summary of the Full Imagery Procedure

Imagery Rescripting of Socially Traumatic Memories in Social Phobia		
Phase 1	Cognitive Restructuring	To look at evidence for and against the encapsulating belief linking the negative image to the socially traumatic memory in order to achieve some belief change.
Phase 2	Imagery Rescripting	To update the socially traumatic memory
Stage 1	The patient relives the event from the age at which it occurred
Stage 2	The patient relives the event from the perspective of their current adult age observing what is happening to their younger self
Stage 3	The patient relives the event from the age at which it occurred again. This time their adult self is with them, may intervene, offer new information, and take a compassionate stance towards the younger self.

**Table 2:** Robert's Evidence for His Encapsulated Belief and His Updated Perspective

Evidence for the Encapsulated Belief (16 years old)	Alternatives With New Information (28 years old)
I go red a lot and it looks odd. People have asked ‘Why do you go so red?’	What I have learned in therapy is that everyone in fact blushes, and it feels a lot redder than how it looks. When I saw myself on video, I looked slightly peachy in color, which was very different to how I thought I would look. My feelings are an unreliable guide to how I look and I am learning to not focus on them because they make me feel more anxious. When I was a teenager, my peers sometimes asked me why I blushed. I have noticed this has not happened to me as an adult. Probably kids pick up on things that are different and maybe they were trying to be funny so they pointed it out. Just because I blush doesn't make me odd, it means I am normal. Everyone blushes.
Other people do presentations in class and I avoid them. Therefore, I am not as good as other people. I am incapable, odd, and a failure.	Everyone gets nervous about presentations. That is so normal. Thinking back, I remember that there were other kids who didn't do them too. I have actually done them now and watched myself on video. I could not see my nervous feelings. If I had the information that I have today about how I come across, I would not have avoided them in school. I am capable of doing them and even if I did not, that does not make me a failure. I did not fail school or university, I just had normal, anxious feelings about public speaking.
My sister's friends rejected me in the canteen when they saw me. I am not popular like she is. I am less than other people expect.	What I know now is that my sister's friends did not actually reject me. I left the canteen before they had a chance to talk to me. I have friends at school, just not as many friends as my sister. But girls are naturally more chatty than boys, so it's understandable that they would have a wider circle of friends. It doesn't make me less than her or anyone else. I do not actually know what other people expect of me. I know I expect others to be friendly and they probably expect similar things of me.

**Table 3:** Megan's Evidence for Her Encapsulated Belief and Her Updated Perspective

Evidence for the Encapsulated Belief (18 years old)	Alternatives With New Information (30 years old)
I blush.	I know now everyone blushes! It is normal and sometimes even endearing. People blush too for lots of different reasons. People blush when they run, when they are hot, when they drink alcohol, or when they feel embarrassed. It is not a sign of being inferior. Plus it is actually not that noticeable. When I saw myself on video, I could barely see the blush, it was certainly a lot less noticeable than I feared it would be. People do not reject others for blushing.
Jeff was critical of me, like how I held my fork.	Get rid of him! He is a jerk. How I hold my fork is certainly not a sign of being inferior. I could eat with my hands and still that would not make me inferior. Inferior is about being unkind, cruel and horrible and that is not what I am.
The bathroom incident	That incident lasted a few minutes and it was not my fault. The way Jeff reacted was unkind. Even though his friend, Neil, had a history of being mean to me, I was kind and did not mention that he had made a mess. That is a sign of being evolved not inferior.
